# Multipotent human stromal cells isolated from cord blood, term placenta and adult bone marrow show distinct differences in gene expression pattern

**DOI:** 10.1016/j.gdata.2014.11.011

**Published:** 2014-11-28

**Authors:** Nicholas Matigian, Gary Brooke, Faten Zaibak, Tony Rossetti, Katarina Kollar, Rebecca Pelekanos, Celena Heazlewood, Alan Mackay-Sim, Christine A. Wells, Kerry Atkinson

**Affiliations:** a Mater Medical Research Institute, University of Queensland, Woolloongabba, Brisbane, 4102, Australia; b Australian Institute for Bioengineering and Nanotechnology, University of Queensland, Brisbane, 4072, Australia; c Eskitis Institute for Drug Discovery, National Centre for Adult Stem Cell Research, Griffith University, Brisbane, 4111, Australia; d The Institute for Infection, Immunity and Inflammation, College of Medical, Veterinary and Life Sciences, The University of Glasgow, Scotland, G12 8TA, UK; e Murdoch Children's Research Institute, The University of Melbourne, Royal Children's Hospital, Parkville, Victoria, 3052, Australia

**Keywords:** Mesenchymal stromal cells, Multipotent human stromal cells, Mesenchymal stem cells

## Abstract

Multipotent mesenchymal stromal cells derived from human placenta (pMSCs), and unrestricted somatic stem cells (USSCs) derived from cord blood share many properties with human bone marrow-derived mesenchymal stromal cells (bmMSCs) and are currently in clinical trials for a wide range of clinical settings. Here we present gene expression profiles of human cord blood-derived unrestricted somatic stem cells (USSCs), human placental-derived mesenchymal stem cells (hpMSCs), and human bone marrow-derived mesenchymal stromal cells (bmMSCs), all derived from four different donors. The microarray data are available on the ArrayExpress database (www.ebi.ac.uk/arrayexpress) under accession number E-TABM-880. Additionally, the data has been integrated into a public portal, www.stemformatics.org. Our data provide a resource for understanding the differences in MSCs derived from different tissues.

**Table:** 

Specifications [*standardized info for the reader*]	
Organism/cell line/tissue	Human primary cell populations from • bone marrow-derived mesenchymal stromal cells (bmMSCs) • placental-derived mesenchymal stromal cells (pMSCs) • human cord blood-derived unrestricted somatic stem cells (USSCs)
Sex	Male and female
Sequencer or array type	Human whole-genome Illumina Human-Ref8 version 2 BeadChips (Illumina, Inc.)
Data format	Both raw and normalized
Experimental factors	Tissue comparison
Experimental features	Very brief experimental description
Consent	Level of consent allowed for reuse if applicable
Sample source location	City, country of model organism and/or latitude and longitude (and GPS coordinates) for collected samples if applicable

## Direct link to deposited data

http://www.ebi.ac.uk/arrayexpress/experiments/E-TABM-880/

http://www.stemformatics.org/datasets/view/6064

## Experimental design, materials and methods

### Isolation and culture of cells

#### USSCs

Cord blood was collected with informed consent from healthy mothers undergoing elective Caesarean section. The protocol was approved by the University of Melbourne and the Royal Women's Hospital Human Ethics Review Committees. A USSC population was successfully generated as described by Kögler et al., 2004. The phenotype of this population has previously been published [1], [2]. Cells from passages 5–8 were used in this study.

#### bmMSCs and pMSCs

Human bone marrow was obtained from healthy donors after informed consent. Placentas were obtained from healthy mothers during routine elective Caesarean section births at or near term. Full informed consent was obtained. The protocols used to obtain bone marrow and placental MSCs have been described [3], [4]. Cells from passages 4–6 were used in this study. Protocols for the isolation and use of each population were approved by the Mater Health Services Human Ethics Review Committee, Brisbane.

### Differentiation assays

#### Osteogenic lineage

Cells were cultured for 21 days in medium containing 10–7 M dexamethasone, 10 mM glycerol-2-phosphate disodium salt (Sigma) and 50 μg/ml ascorbic acid 2 phosphate (Sigma). Cells were fixed for 10 min with 70% ice-cold ethanol at 4 °C and stained with 1% Alizarin Red S (Sigma) in distilled water, pH 4.2. After cells were washed in distilled water and a final wash with PBS (Ca^2 +^ and Mg^2 +^ free), images were captured using a Leica DMIRB inverted microscope and AxioVision 4.2 software (Carl Zeiss AG, www.zeiss.com).

#### Adipogenic lineage

Cells were cultured in medium containing DMEM, 1 μM dexamethasone (Sigma), 5 μg/ml insulin (Sigma), 60 μM indomethacin (Sigma) and 0.5 mM 3-isobutyl-1-methylxanthine (IBMX; Sigma) for 14 days. Adipogenic differentiation was assessed by staining cells with Oil Red O (Sigma).

### Flow cytometry

To detect the presence of cell surface antigens, cells were washed in PBS and detached from flasks using TrypLE Select (Invitrogen). Cells were incubated for 20 min at 4 °C with monoclonal antibodies to CD29, CD31, CD34, CD44, CD45, CD49d, CD49e, CD50, CD73, CD90, CD105, CD146, CD166, GD2, Stro-1, SSEA-4, TRA-1-60 and TRA-1-81, CCR1, CCR3, CCR5, CCR8, CCR10, CCR11, CXCR3, and CXCR4 (BD Biosciences). Flow cytometry analysis was performed on an LSR II (Becton Dickinson) and analyzed using FCS Express software (De Novo, www.denovosoftware.com). To detect the presence of intracellular chemokine receptors, cells were fixed in 4% paraformaldehyde in PBS for 10 min and washed in staining buffer (300 × *g*, 5 min, 4 ° C) before being permeabilized with Fix/Perm buffer (eBioscience, California, USA) for 30 min at 4 °C in the dark. This solution was removed and the permeabilized cells were then stained for the intracytoplasmic expression of chemokine receptors using the antibodies listed above.

### Gene expression profiling

#### RNA extractions

Total RNA was extracted at passages 4–6 from human bmMSCs, pMSCs and USSCs, using 4 separate donors for each. RNA was extracted using a Qiagen RNeasy kit (www.qiagen.com). All RNA preparations were quantified using a Nanodrop spectrophotometer (Thermo Scientific) and quality was accessed using an Agilent 2100 Bioanalyser (RNA Nano chips). The RNA integrity number ranged between 9.9 and 10, demonstrating high quality starting material.

#### Sample labeling and scanning

Five hundred nanograms of RNA was amplified using the Ambion Illumina RNA amplification kit with biotin UTP labeling (Ambion, Inc), including a 4 h in vitro transcription using T7 RNA polymerase. A total of 750 ng of cRNA was hybridized to human whole-genome Illumina Human-Refseq8 v2 BeadChips (Illumina, Inc.). Slides were scanned on an Illumina Beadstation and bead summarization was performed using BeadStudio Version 3.1.7 (Illumina, Inc). The microarray data are available on the ArrayExpress database (www.ebi.ac.uk/arrayexpress) under accession number E-TABM-880. Additionally, the data have been integrated into a public portal, Stemformatics [5]. Here all the microarray data can be visualized and compared to 100 + other stem cell datasets (http://www.stemformatics.org).

#### Data normalization and filtering

Data were exported from BeadStudio with no additional processing, and imported to R/BioConductor using the readBead function from the BeadExplorer package. Background adjustment and quantile normalization was performed using function: bg.adjust and normalize.quantiles. Genes were initially filtered using Illumina® detection *p*-value. A gene/probe was included in QC assessment if it had a detection *p*-value ≥ 0.99 all four donor samples within the tissue source.

## Results

### Cell surface antigen phenotyping

Based on a standard panel of MSC-descriptive antibodies, all the lines were virtually identical. All expressed CD29, CD44, CD49d, CD49e, CD73, CD90, CD105, CD146, CD49e and CD166 (Table 1). All three cell populations were negative for Stro-1, CD45, CD34, CD50, CD106, and the pluripotency markers SSEA-4, TRA-1-60 and TRA-1-81 (Table 1).

### Chemokine receptor display

The surface and intracellular chemokine displays of bmMSCs and pMSCs were very similar. USSCs differed from bmMSCs and pMSCs by showing positive staining for the presence of intracellular CCR8, CCR10 and CCR11 (Table 2).

### Mesodermal differentiation assays

**Fig. 1:**
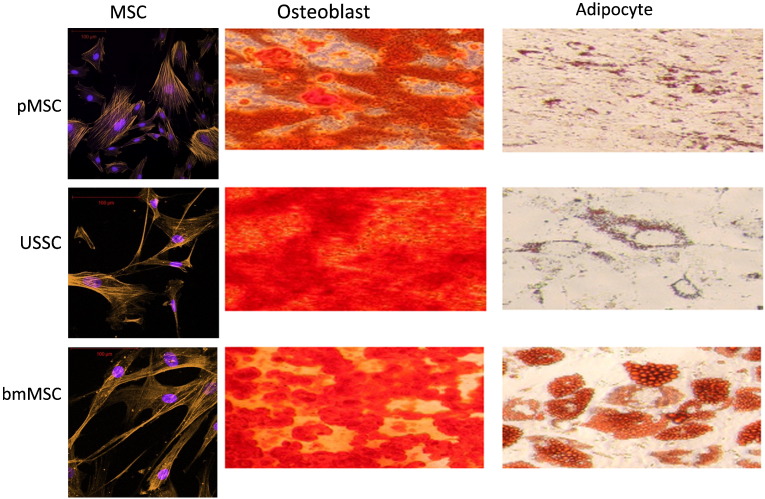
Mesodermal differentiation by MSCs from different sources. Left column: undifferentiated MSCs have a fibroblast-like phenotype, actin stained with phalloidin, and nuclei stained with DAPI. Middle column: calcium-rich matrix produced by osteoblasts is stained with Alizarin Red; right column: lipid droplets in adipocytes were stained with Oil Red O. Rows from top to bottom: MSCs sourced from term placenta (pMSCs), cord blood (USSCs) and bone marrow (bmMSCs). Magnification × 40.

Typical MSC morphology was confirmed in culture (Fig. 1), and the mesodermal differentiation potential of each of the three cell populations was assessed in vitro to determine their multipotency ability. This was analyzed according to the cells' ability to differentiate into osteocytes and adipocytes. All three undifferentiated populations showed marked ability to differentiate to the osteogenic lineage. bmMSCs showed stronger differentiation to the adipogenic pathway that pMSCs and USSCs, each of which showed only a slight degree of adipogenesis (Fig. 1).

### Gene expression quality

**Fig. 2:**
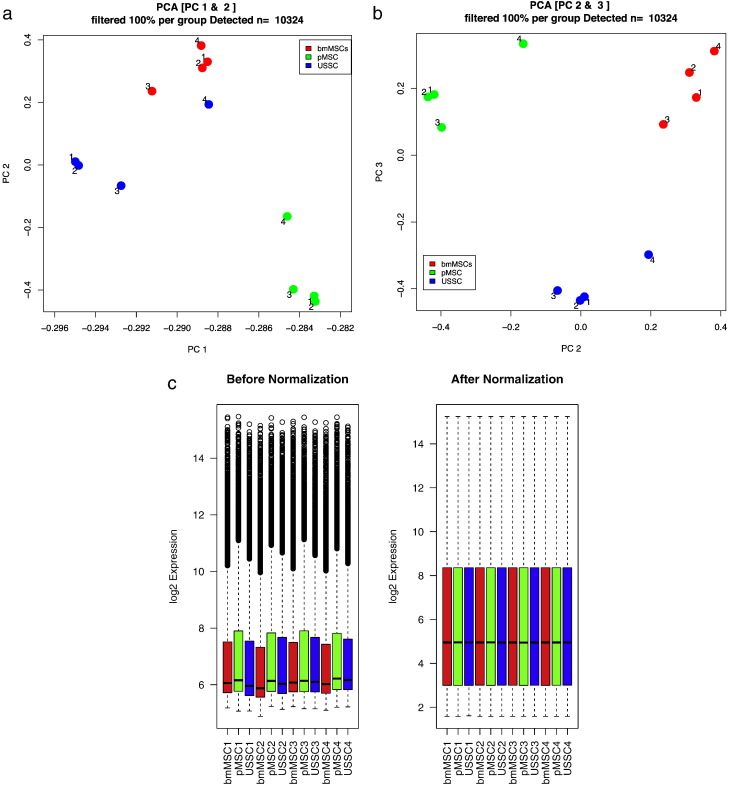
QC and principal component analysis of gene expression data (detected probes only): Panel a: comparing components 1 (89.1% variance) and 2 (4.1% variance); b: comparing components 2 (4.1% variance) and 3 (2.3% variance). Legend: MSC source indicated by color: pMSCs (green); bmMSCs (red); USSCs (blue). c: Range of gene expression and median values in samples pre (left) and post (right) normalization.

Normalization reduced the between array variation (Fig. 2). Principle component analysis was performed to demonstrate the difference between the MSC sources and the reproducibility of the replicate donors. When plotting on the first two components, the samples clustered based on their tissue source, but some donor variation was apparent (Fig. 2a). All replicates were tightly clustered except for one USSC sample (Chip ID: 4294077038_D; Sample ID: USSC4). However, this sample was still highly correlated to the other USSC donors (Average Pearson of 0.89) compared to 0.95 for the bmMSCs and pMSCs. When plotting components two and three the samples clustered based on their tissue source (Fig. 2b).

## Author contributions

N. Matigian performed the microarrays and contributed to the bioinformatics analysis and to the writing of the manuscript. G. Brooke co-initiated the study and supervised the isolation of the bone marrow MSCs and the placental MSCs and the flow cytometry and mesodermal differentiation of the cells. F. Zaibak and R. Williamson generated the unrestricted somatic stem cells. T. Rosetti, K. Kollar, R. Pelekanos and C. Heazlewood isolated the bone marrow MSCs and the placental MSCs and generated the flow cytometry data and mesodermal differentiation data. A. Mackay-Sim, C. Wells and K. Atkinson co-funded the study. C. Wells supervised the microarray data acquisition and the bioinformatics analysis and contributed to the writing of the manuscript. K. Atkinson wrote the initial draft and final version of the manuscript.

## Figures and Tables

**Table 1:** Cell surface markers on bmMSCs, pMSCs and USSCs.

Marker association	CD number/name	Other name	bmMSC	hpMSC	USSC
Mesenchymal stromal cell-associated markers	CD29	Integrinβ1	Positive	Positive	Positive
	CD44	HCAM (homing cell adhesion molecule)	Positive	Positive	Positive
	CD73	Ecto-5′-nucleotidase	Positive	Positive	Positive
	CD90	Thy-1	Positive	Positive	Positive
	CD105	Endoglin	Positive	Positive	Positive
	CD146	MCAM (melanoma cell adhesion molecule)	Positive	Not done	Positive
	GD2	Neural ganglioside 2	Faint staining	Faint staining	Faint staining
Mesenchymal stem cell-associated marker	Stro-1	Stromal cell molecule-1	Negative	Negative	Negative
Endothelial marker	CD31	PECAM-1 (platelet/endothelial cell adhesion molecule)	Negative	Negative	Negative
Hematopoietic cell-associated markers	CD34	Mucosialin	Negative	Negative	Negative
	CD45	Leukocyte common marker	Negative	Negative	Negative
Other adhesion molecules	CD49d	VLA-4 (very late antigen-4)	Positive	Positive	Positive
	CD49e	VLA-5a (very late antigen-5a)	Positive	Positive	Positive
	CD50	ICAM-3 (intercellular adhesion molecule)	Negative	Faint staining	Negative
	CD166	ALCAM (activated leukocyte cell adhesion molecule)	Positive	Positive	Positive
Pluripotency markers	SSEA-4	Stage-specific embryonic antigen-4	Negative	Negative	Negative
	TRA 1-60	TRA (Tumor Rejection Antigen)	Negative	Negative	Negative
	TRA 1-81	TRA (Tumor Rejection Antigen)	Negative	Negative	Negative

**Table 2:** Chemokine receptor display by bmMSCs, hpMSCs and UCCSs.

Chemokine receptor	bmMSC surface display	bmMSC intracellular display	hpMSC cell surface display	hpMSC intracellular display	USSC cell surface display	USSC intracellular display
CCR1	Faint staining	Positive	Faint staining	Positive	Negative	Faint staining
CCR3	Negative	Positive	Negative	Positive	Negative	Positive
CCR5	Negative	Negative	Negative	Negative	Negative	Negative
CCR8	Faint staining	Negative	Faint staining	Negative	Negative	Positive
CCR10	Negative	Negative	Negative	Negative	Negative	Positive
CCR11	Faint staining	Negative	Faint staining	Negative	Negative	Positive
CXCR3	Negative	Positive	Negative	Positive	Negative	Positive
CXCR4	Positive	Positive	Positive	Positive	Negative	Positive
